# Immunohistochemical characterization of stem cell and differentiation markers of the dental pulp of human natal teeth

**DOI:** 10.4155/fsoa-2018-0062

**Published:** 2018-10-04

**Authors:** Heeresh Shetty, Adesh Kakade, Shishir Shetty, Prasanna Neelakantan, Saurabh Nagar, Rajiv S Desai, Kavita Beri

**Affiliations:** 1 Department of Conservative Dentistry & Endodontics Nair Hospital Dental College, Mumbai, India; 2 Department of Pediatric Dentistry, Nair Hospital Dental College, Mumbai, India; 3 Department of Conservative Dentistry & Endodontics, A.B Shetty Institute of Dental Sciences, Mangalore, India; 4 Discipline of Endodontology, Faculty of Dentistry, University of Hong Kong, Hong Kong; 5 Department of Oral & Maxillofacial Pathology, Nair Hospital Dental College, Mumbai, India; 6 Biomedical Engineering, Rutgers University of New Jersey, New Brunswick, NJ, 08854, USA; 7 Center for Dermal Research, NJ Center for Biomaterials Rutgers, The State University of New Jersey, NJ, 07302, USA

**Keywords:** CD44, dental pulp stem cell (DPSC), desmin, immunohistochemical expression, natal teeth, nestin, Oct-4, osteopontin and myogenin, SOX2

## Abstract

**Aim:**

Dental pulp stem cells, which are primarily derived from the pulp tissues of human teeth, have rarely been obtained from natal teeth. This study investigated the stem cell and differentiation markers of the dental pulp of natal teeth using immunohistochemistry.

**Materials & Methods:**

The pulp tissue from extracted natal teeth (n = 2) of a 20-day-old healthy male was examined for immunohistochemical expression of stem cell (Oct-4 and SOX 2) and differentiation markers (Nestin, CD 44, desmin, osteopontin and Ki- 67).

**Results:**

The pulp tissue of the natal teeth expressed immunopositivity for nestin, CD 44 and SOX2.

**Conclusion:**

Natal teeth, if preserved properly, could serve as sources of dental pulp stem cells that are an improvement on deciduous teeth.

## Lay abstract

Natal teeth are present at birth. They are usually removed prophylactically to avoid the complications such as aspiration, ulcerations on the tongue and injuries to the mother during feeding. To study discarded natal teeth as a potential stem cell source, immunohistochemistry was used to check for the expression of stem cells and markers on the dental pulp of the natal teeth. This was compared with the stem cell potential of deciduous teeth. Natal teeth exhibited higher immunohistochemical expression, which suggests that discarded tissues like natal teeth are a potential source for stem cells.

The first tooth erupts in the human oral cavity at 6 months of age. However, an occasional entity termed ‘natal teeth’ may be present in some babies at birth. When these teeth erupt within the first month of birth, they are termed neonatal teeth [1]. Although the etiology of early eruption of these teeth is still unknown, the incidence of natal and neonatal teeth ranges between 1:2000 and 1:3500 [2,3]. Although these teeth are not necessarily considered pathological or abnormal, they may lead to several complications including aspiration, swallowing due to premature exfoliation, ulcerations on the tongue and lips as well as injuries to the mother during feeding [4,5]. To avoid the aforementioned complications, these teeth are prophylactically removed.

Dental pulp stem cells (DPSCs) are mainly isolated from the pulp tissue of exfoliated deciduous teeth, deciduous incisors or permanent third molars [6,7]. There have been case reports to show that supernumerary teeth and natal teeth may serve as potential sources of DPSCs [8–10]. Despite being uncommon, natal teeth are seen just after the developmental stage, where the stem cells are in the peak of their activity. Therefore, these teeth may be explored as a source of stem cells, similar to the embryonic sac and the embryonic fluid. However, the stem cell and differentiation marker expression profile of natal cells, in comparison with deciduous teeth, is unknown. The aim of this study was to investigate the expression of stem cell/progenitor cell and differentiation markers of natal teeth pulp tissue, compared with pulp tissue obtained from a deciduous tooth, using immunohistochemistry.

## Materials & methods

Two natal teeth of a 20-day-old healthy male patient and a deciduous mandibular first molar of an 11-year-old healthy male patient were extracted under local anesthesia based on a protocol approved by the Institutional Review Board and Ethics Committee. Informed consent was obtained from the parents of these patients to extract the teeth and use them for further analysis. The natal teeth were extracted for prophylactic reasons as outlined previously, while the deciduous teeth were extracted due to mobility. The deciduous molar served as the positive control. Following extraction, the teeth were immediately fixed in 10% neutral buffered formalin for 24 h. The dental pulp tissue was removed from the deciduous mandibular first molar by splitting the tooth with a chisel and immediately fixing in 10% neutral buffered formalin for 24 h.

### Histologic procedures

After fixation, the natal teeth were demineralized in 10% EDTA (pH = 7.4) for 6 months at room temperature. Subsequently, the samples were rinsed under running water for 4 h followed by dehydration with ascending concentrations of ethanol. Then, the teeth were deparaffinized in xylene, infiltrated, and embedded in paraffin. With the microtome set at 5 μm, longitudinal serial sections were cut and stained with hematoxylin–eosin.

### Immunohistochemical procedures

Serial sections, 5 μm in thickness, prepared from archived formalin-fixed, paraffin-embedded (FFPE) tissue blocks, were used for immunohistochemical analysis. Sections were deparaffinized and rehydrated following standard methods. Briefly, the sections were deparaffinized three-times with xylene for 5 min and rehydrated in graded ethanol (80–100%) for 5 min. Antigen retrieval was performed by boiling at 98°C for 40 min in 0.01 mol/l sodium citrate buffer (pH 6.0). Details of the primary antibody clones, sources and titers are listed in Table 1.

**Table 1.** **Details of the primary antibody clones, sources and titers.**

**Primary serum**	**Clone**	**Source**	**Working titer**
OCT4	ab18976	Abcam Cambridge, MA, USA	1:100

SOX2	Ab97959	Abcam Cambridge, MA, USA	1:100

CD44 (HCAM)	Ab-4	Thermo Fisher Scientific, Fermount, CA, USA	1:150

Nestin	ab93666	Abcam Cambridge, MA, USA	1:120

Osteopontin	AKm2A1	Santa Cruz Biotechnology Inc., Dallas, TX, USA	1:50

Desmin	D33	Thermo Fisher Scientific, Fermount, CA, USA	Prediluted

Myogenin	F5D	Thermo Fisher Scientific, Fermount, CA, USA	Prediluted

CD34	C-18	Santa Cruz Biotechnology Inc, Dallas, TX, USA	1:150

The endogenous peroxidase activity was blocked and the tissue samples were incubated with OCT4, SOX2, CD44, nestin, osteopontin, desmin and myogenin antibodies for 90 min. Subsequently, the slices were rinsed and incubated with the biotinylated secondary antibody at room temperature for 30 min. The bound antibody complexes were stained for 3–5 min or until appropriate for microscopic examination and then counter stained with hematoxylin (30 s) and mounted.

The expression of OCT4, SOX2, CD44, nestin, osteopontin, desmin, myogenin and CD34 on the natal as well as the deciduous tooth (positive control) tissue samples were analyzed. The immunoreactivity of the specimens was interpreted based on the intensity of the staining. Score ranks usually lie in a range from ‘negative’ (mostly marked as ‘-’) to ‘positive’, which was signed with different amount of ‘+’ depending upon the intensity of the stain. The samples were examined with conventional light microscope. For OCT4, SOX2, osteopontin, myogenin nuclear immunohistochemical staining was considered positive whereas for nestin and desmin cytoplasmic immunohistochemical staining was considered positive. Membranous immunohistochemical staining was considered positive for CD34 and CD44. The staining intensity was graded as (+) weak, (++) moderate and (+++) intense according to the overall appearance at different powers of magnification, in other words, 4×, 10× and 40×, respectively and (-) for no staining.

## Results

### Histological & immunohistochemical observations

**Figure 1.**
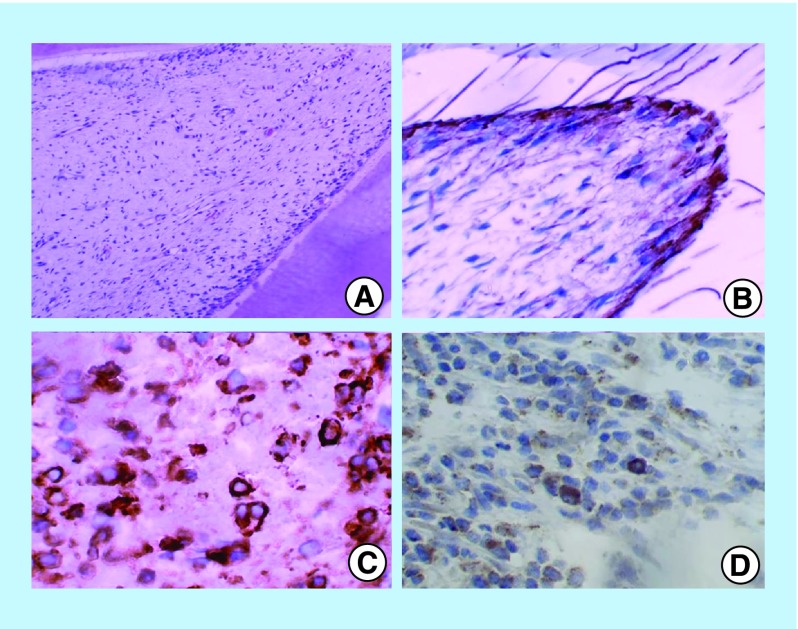
**Photomicrograph of the natal tooth.** Figure shows: **(A)** Hematoxylin and eosin-stained section [10× magnification]; **(B)** Strong immunohistochemical expression of Nestin, restricted to odontoblasts (40× magnification); **(C)** Strong immunohistochemical expression of CD44, restricted to odontoblasts and pulp tissue (40× magnification); **(D)** Strong expression of SOX2 in pulp tissue (40× magnification).

**Figure 2.**
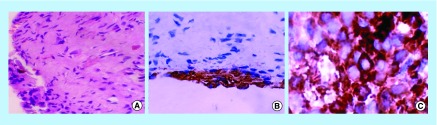
**Photomicrograph of the deciduous tooth (control).** **(A)** Hematoxylin and eosin-stained section (40× magnification); **(B)** Immunohistochemical expression of CD44 in peripheral odontoblasts (40× magnification); **(C)** Immunohistochemical expression of nestin, restricted to the peripheral odontoblasts (40× magnification).

Hematoxylin and eosin stained sections of the tissues obtained from the natal and deciduous teeth showed myxomatous connective tissue with blood vessels and inflammatory cells. A clear odontoblastic lining was evident adjacent to the secondary dentin (Figures 1A & 2A).

Immunohistochemical examination (Table 2) demonstrated that both the natal and deciduous teeth expressed nestin and CD44 immunopositivity for odontoblasts and connective tissue. However, expression of all the markers was stronger in the natal teeth than the deciduous tooth. In contrast, SOX2 was expressed only by natal teeth. Remaining immunohistochemical markers such as osteopontin, desmin, myogenin, OCT4 and CD34 were negative in both the teeth.

**Table 2.** **Immunohistochemical grading of stem cell differentiation markers.**

**Markers**	**N1**	**N2**	**D (control)**
OCT4	†	†	†

SOX2	^‡^	‡	†

CD44	^§^	‡	‡

Nestin	^§^	§	‡

Osteopontin	^†^	†	†

Desmin	^†^	†	†

Myogenin	^†^	†	†

CD34	^†^	†	†

^†^No immunostaining.

^‡^Moderate.

^§^Intense.

D: Deciduous molar; N: Natal tooth.

## Discussion

Stem cells are an excellent source of generation of a huge number of mature cells through a sequential procedure of proliferation and differentiation, retaining their ability to self renew to maintain the stem cell pool. Stem cell markers are the protein products that identify a multipotent single cell capable of recapitulating. There are four types of cell: embryonic stem cell (ESC), hematopoietic stem cell (HSC), mesenchymal/stromal stem cell (MSC/SSC) and neural stem cell (NSC).

The purpose of this study was to characterize the stem cellness of the dental pulp obtained from natal teeth, which are in general considered waste teeth, using histological and immunohistochemical methods. Only two studies thus far have successfully isolated and characterized human natal DPSCs (hNDPSCs) *in vitro* [9,10]. Although Karaoz *et al*. [9] demonstrated the expression of ESC markers (OCT4, REX1, FOXD3, SOX2 and Nanog) and differentiation potential of DPSCs derived from natal teeth toward adipogenic, chondrogenic, osteogenic, myogenic and neurogenic lineages, Suchanek *et al*. [10] demonstrated successful *in vitro* differentiation of hNDPSCs into induced pluripotent cells that were morphologically similar to pancreatic islet cells.

Prior to investigating the multilineage differentiation potential of DPSC, an overview of marker expression in basic pulp tissue is crucial. In the present study, for the first time, we demonstrate the immunohistochemical characterization of stem cell and differentiation markers in FFPE tissue sections of natal teeth. OCT4 and SOX2 serve as reliable markers for identifying the NSC population. OCT4 is an ESC marker, identified as a DNA-binding protein that activates gene transcription via an octamer motif [11]. OCT4 expression is required to sustain stem cell self-renewing capacity and pluripotency. SRY–box2, also known as SOX2 is a transcription factor, known to be expressed at high levels in the neuroepithelium of the developing CNS, and is considered to be critical for NSC proliferation and differentiation [12]. Previous studies have identified that the stem cell or potential progenitor cell population in the dental pulp constitutes <1 % of the total cells [13,14]. This may be attributed to the nonexpression of stem cell markers (OCT4 and SOX2) in the deciduous pulp (positive control in this study). The present study showed positive immune expression of SOX2 in the dental pulp of natal teeth, which confirms the presence of higher percentage of stem/progenitor cell population compared with the deciduous pulp (Figure 1D).

Expression of CD44, a positive MSC marker and CD34, a negative MSC marker were also analyzed in this work. CD44, a ubiquitous, multistructural, multifunctional cell surface glycoprotein is involved in cell-to-cell adhesion, cell-matrix interactions and cell migration. It is widely expressed in several cell types. CD44 is expressed at the time of extracellular matrix formation in many sites during embryonic development. CD44, a cell-adhesion factor, is involved in the induction of mineralization in the dental pulp. Interestingly, it was overexpressed in the natal tooth specimen when compared with the control (Figures 1C & 2B). CD34 is an HSC marker expressed on a small fraction of human bone marrow cells. Since the CD34 positive enriched cell population from the bone marrow is responsible for most of its hematopoietic activity, it is considered to be one of the most critical markers of HSCs. This study showed immunonegativity of CD34 in the natal as well as the control deciduous molar, thus corroborating with the findings of Karaoz *et al*. [9].

Nestin, a class VI intermediate filament protein, is predominantly expressed in the CNS [15,16]. Although nestin does not form intermediate filaments by itself, it co-assembles with vimentin or alpha-internexin to form a heterodimer coiled-complex, which are intermediate filaments. Its transient expression has been suggested to be a major step in neural differentiation. However, it is also expressed in non-NSC populations such as pancreatic islet progenitors and hematopoietic progenitors and DPSCs. In young developing human deciduous tooth germs, nestin immunoreactivity has been reported in the odontoblast, pulp fibroblasts, odontoblastic processes up to the dentinoenamel junction and pulp cells. In mature teeth, expression of nestin appears to be restricted to odontoblasts. The findings of the present study are in agreement with previous findings (Figures 1B & 2C) [17].

Natal teeth provide a unique population of stem cells that can be attributed to their entirely intrauterine development. They are different from deciduous and permanent teeth in their structure as well as function. In their study on exfoliated deciduous teeth, Miura *et al*. demonstrated a significant difference between SHED and DPSCs in terms of rates of proliferation, cell population doublings, sphere-like cell cluster formation and osteoinductive capacity, all of which were better in SHED. Our study, which compared natal teeth and deciduous teeth, also found these teeth as an excellent and abundant source of stem cells.

An interesting finding in the present study that differed to the results of Karaoz *et al*. is the lack of expression of myogenic (desmin and myogenin) and osteogenic differentiation markers (osteopontin). This may be attributed to the fact that the above-mentioned study used cell culture techniques, in contrast to our report, which used FFPE tissues. Our study provides evidence that natal teeth have shown very strong positive expression of CD44 (MSC), SOX2 (ESC) and Nestin (NSC) markers. Natal teeth therefore may be a viable source of stem cells to induce repair or regeneration of tissue, wherever applicable and for treatment of degenerative tissues. To the best of our knowledge, this is the first study to demonstrate positive immunoexpression of various stem cell markers in FFPE sections of a usually discarded tooth such as the natal tooth.

## Conclusion

Taken together, the results of this study support the use of natal teeth as a potential source of pluripotent stem cells for future cell-based therapies.

## Future perspective

Since the natal teeth develop during the intrauterine stages, the stem cells of natal teeth originate at much earlier stages of development and exhibit excellent potency to differentiate into various structures. This property could be utilized for treating various clinical conditions. They will also be useful in dentin tissue engineering, teeth replantation and pulp revascularization. However, the stem cells of natal teeth origin have been heretofore underestimated due to the sparse available literature and hence future research in this direction is warranted to use them to their maximal potential.

**Summary points**

- The immunohistochemical expression of OCT4, SOX2, CD34, CD44, nestin, osteopontin, desmin, myogenin and KI67 on the dental pulp of natal as well as the deciduous tooth tissue samples was analyzed.
- The natal teeth as well as the deciduous tooth specimens expressed immunopositivity for nestin and CD44. However, the natal teeth specimen showed an overexpression of these markers compared with its counterpart.
- SOX2, a transcription factor considered to be critical for neural stem cell proliferation and differentiation, was expressed only by the natal teeth.
- Natal teeth, a usually discarded tissue, can be considered as a potential source of pluripotent stem cells for future cell-based therapies.
